# MRI DTI and PDFF as Biomarkers for Lower Motor Neuron Degeneration in ALS

**DOI:** 10.3389/fnins.2021.682126

**Published:** 2021-08-26

**Authors:** Thorsten Lichtenstein, Alina Sprenger, Kilian Weiss, Nils Große Hokamp, David Maintz, Marc Schlamann, Gereon R. Fink, Helmar C. Lehmann, Tobias D. Henning

**Affiliations:** ^1^Institute for Diagnostic and Interventional Radiology, Faculty of Medicine and University Hospital Cologne, University of Cologne, Cologne, Germany; ^2^Department of Neurology, University Hospital of Cologne, University of Cologne, Cologne, Germany; ^3^Philips Healthcare, Hamburg, Germany; ^4^Cognitive Neuroscience, Institute of Neuroscience and Medicine (INM-3), Research Centre Jülich, Jülich, Germany; ^5^Department of Neuroradiology, Center Hospital Luxembourg, Luxembourg City, Luxembourg

**Keywords:** amyotrophic lateral sclerosis, diffusion tensor imaging, proton density fat fraction, neurodegeneration, motor neuron disease

## Abstract

**Objective:**

To evaluate the utility of nerve magnetic resonance imaging (MRI), diffusion tensor imaging (DTI), and muscle MRI multi-echo Dixon for assessing lower motor neuron (LMN) degeneration in amyotrophic lateral sclerosis (ALS).

**Methods:**

In this prospective observational cohort study, 14 patients with ALS and 13 healthy controls underwent a multiparametric MRI protocol, including DTI of the sciatic nerve and assessment of muscle proton density fat fraction of the biceps femoris and the quadriceps femoris muscles by a multi-echo Dixon sequence.

**Results:**

In ALS patients, mean fractional anisotropy values of the sciatic nerve were significantly lower than those of healthy controls. The quadriceps femoris, but not the biceps femoris muscle, showed significantly higher intramuscular fat fractions in ALS.

**Interpretation:**

Our study provides evidence that multiparametric MRI protocols might help estimate structural nerve damage and neurogenic muscle changes in ALS.

## Introduction

Amyotrophic lateral sclerosis (ALS) is a neurodegenerative disease with a variable phenotype that primarily affects the motor system. Differences in phenotype include deviating affection patterns of the upper motor neuron (UMN) or lower motor neuron (LMN) and disease progression differences. This variability causes difficulties in monitoring the course of the disease. Especially when conducting research (e.g., to evaluate new therapies), the lack of sensitive methods to detect short-term changes is one of the critical challenges (Turner et al., 2009; Berry and Cudkowicz, 2011; Simon et al., 2015). Since damage to LMN is decisive for disease progression in ALS (Simon, 2016), it seems particularly promising to detect its degeneration as precisely as possible. So far, disease progression has been monitored using electrophysiological and clinical tests [in particular motor nerve conduction studies (mNCS) and motor unit number estimation (MUNE)] (Carvalho and de Swash, 2016). However, these clinical tests suffer from several limitations. For example, they depend on UMN function and the patient’s overall clinical condition (Simon et al., 2014).

Magnetic resonance imaging (MRI) has the potential to become a valuable, non-invasive biomarker for neurodegeneration in ALS. Previously, MRI applications to ALS mainly focused on structural alterations of the ALS patients’ brain and spinal cord. Numerous studies using diffusion tensor imaging (DTI) demonstrated degeneration of the corticospinal tract and motor-associated brain regions. Most studies demonstrated a decrease of DTI-based fractional anisotropy (FA), a marker for axonal degeneration and demyelinating conditions (Jende et al., 2020, 2021), which indicates an involvement of the UMN (Ellis et al., 1999; Toosy et al., 2003; Iwata et al., 2008; Blain et al., 2009; Metwalli et al., 2010; Cirillo et al., 2012; Bede et al., 2015).

In contrast, data about the MRI’s utility to detect LMN degeneration in ALS are sparse (Grolez et al., 2016; Verber et al., 2019). Simon and colleagues recently demonstrated in 19 ALS patients reduced FA in tibial and peroneal nerve segments with subsequent decline at 6-months. These changes correlated with the ALS revised functional rating scale (ALSFRS-R) (Simon et al., 2017).

There is also only limited information about the MRI utility to monitor neurogenic muscle atrophy in ALS. T2-weighted whole-body muscle MRI shows increased relative T2 signal in most limb muscles of ALS patients, as recently reported by Jenkins and colleagues (Jenkins et al., 2018). Clinical and experimental studies indicate that denervated muscles display a reversible high T2 signal as early as 48 h after nerve injury (West et al., 1994; Bendszus et al., 2002). However, these changes are only small and decline with prolonged denervation (Zhang et al., 2008). Human studies also demonstrated that T2 hyperintensity does not correlate well with MR-neurographic parameters (Schwarz et al., 2015).

Recently, Klickovic and colleagues demonstrated in a cohort of 20 ALS patients the feasibility of quantifying muscle fat fraction using the MRI 3-point Dixon technique. They found higher fat fractions in ALS patients’ calf muscles compared to controls (3.34 vs. 1.92%), which correlated well with functional scales (Klickovic et al., 2019).

We have established a multiparametric MRI imaging paradigm that allows simultaneous quantification of nerve injury and intramuscular fat fraction in lower limbs (Lichtenstein et al., 2017). Based on DTI scans of nerves and multi-echo Dixon MRI of adjacent muscles, this protocol detects structural nerve damage and neurogenic intramuscular fat accumulation in different neuropathic conditions. We hypothesized that this protocol might also be suitable for monitoring LMN degeneration in ALS and, therefore, conducted this exploratory study in ALS patients.

## Materials and Methods

### Patients and Healthy Controls

Fourteen patients (6 female, 8 male, mean age 62 ± 6 years) with ALS and 13 healthy controls (6 female, 7 male, mean age 56 ± 9 years) participated in this study. All patients were diagnosed at the Department of Neurology, University Hospital of Cologne, Cologne, Germany, based on the El Escorial Criteria (Ludolph et al., 2015). Patients with neuropathies and contraindications against MRI were excluded. Healthy controls were defined as individuals without anamnestic and clinical signs of polyneuropathy. All patients were on antiglutamate therapy with riluzole (50 mg twice a day). The local Ethics Committee approved the study, and all subjects gave written informed consent before inclusion. All patients received a standard clinical and electrophysiological assessment. The clinical investigation included a complete neurological examination. Besides, the ALSFRS-R was collected for each patient. Briefly, the ALSFRS-R summarizes physical impairment in activities of daily living for a patient with ALS and is used for measuring the progression of the disease (Cedarbaum et al., 1999). It is a revision of the ALSFRS that includes additional questions related to respiratory symptoms. The score includes 12 questions concerning physical functions (e.g., speaking, swallowing, walking) ranging from 0 (severe impairment) to 4 (no impairment) with a maximum score of 40 and a minimum score of 0. For electrophysiological examinations, standard nerve conduction studies were performed. The right tibial and peroneal nerves were used to measure the motor nerve conduction velocity (mNCV), the proximal and distal compound muscle action potential (CMAP), and distal motor latencies (DML). The right sural nerve was used to measure the sensory nerve conduction velocity (sNCV) and the sensory nerve action potential (SNAP). Standard electromyography (EMG) of the tibialis anterior, quadriceps, and biceps femoris muscles was performed using conventional EMG equipment and concentric needle electrodes.

### MRI Protocol

All subjects were examined using an MRI protocol already established in patients with chronic inflammatory polyneuropathy for examining the sciatic nerve and the thigh muscles (Lichtenstein et al., 2017; Schneider et al., 2019). The examinations were performed on a 3T whole-body MRI system (Ingenia, Philips Healthcare, Best, Netherlands). As in the other studies, the patients were positioned in a supine position with feet first. The subjects’ right thigh was examined deep inside a knee coil (dStream T/R Knee 16ch Coil, Philips Healthcare, Best, Netherlands) so that the coil center was located approx. 5–10 cm above the upper pole of the patella.

#### Planning Sequence

To delineate the nerve, a special orientation of a SHINKEI-based three-dimensional T2-weighted turbo spin echo (3D T2 TSE) sequence with fat and vascular signal suppression was used (Cervantes et al., 2015; Kasper et al., 2015; Kollmer et al., 2015). The exact parameters were: TR = 2,000, TE = 273, matrix size 216 × 143 × 143, resolution 1.25 × 1.25 × 0.7 mm^3^, scan duration 2:30 min.

#### T2-Weighted, mDixon TSE Sequence

The anatomical assessment was performed in a transversal, perpendicular to the sciatic nerve, T2-weighted mDixon TSE (2D T2 TSE) sequence. The parameters were: TR = 2,500 ms, TE = 60 ms, matrix size 640 × 468, 30 slices with 4 mm slice thickness and no interslice gap, resolution 0.3 × 0.4 × 4 mm^3^, scan duration 5 min.

#### DTI

A DTI sequence based on single-shot echo-planar imaging was planned in the same way as the 2D T2 TSE. Sequence parameters were: TR = 6500 ms, TE = 62 msec, matrix size 128 × 130, 20 layers with 4 mm layer thickness and without kerf, resolution 1.5 × 1.5 × 4 mm^3^, *b*-values of 0 s/mm^2^ and 800 s/mm^2^, in 20 directions, SENSE factor of 2, scan duration 9:00 min.

#### PDFF

A third, transversely recorded sequence, i.e., a six-echo multi-echo gradient echo sequence (mDixon Quant, Philips Healthcare, Best, Netherlands) generating proton density fat fraction (PDFF) maps, was similarly used for intramuscular fat quantification of the quadriceps femoris (QFM) and biceps femoris muscles (BFM). The parameters were as follows: TR = 10 ms, 6 echoes (TE1 = 1.45 ms, ΔTE = 1.1 ms), matrix 108 × 107 × 4 mm^3^, voxel size 1.8 × 1.8 × 4 mm, 20 slices, flip angle 3° (to minimize T1 bias effects), recording time 1:05 min.

#### Data Analysis

A senior radiologist (T.L.) evaluated the MR images. Measurements were validated by a second senior radiologist (N. G. H.) based on independent assessment of a subset of study participants. The post-processing of the DTI raw data and the complete MRI analysis was performed with IntelliSpace Portal (IntelliSpace Portal 10.0, Philips Healthcare, Best, Netherlands).

To analyze the sciatic nerve in the DTI sequence, six subtotal freehand ROIs were drawn in six adjacent layers of color-coded fractional anisotropy images in correlation with the anatomical information of the *b* = 0 and 2D T2 TSE images. The average FA values of the six slices were then remeasured to obtain each subject’s final FA value. Fiber tracking of the nerve was performed for illustration.

In the PDFF maps, freehand subtotal ROIs were drawn on the three most proximal slices into each part of the quadriceps femoris muscle (vastus lateralis, intermedius, medialis, rectus femoris) and into the short and long heads of the biceps femoris muscle for determination of the average intramuscular fat fraction. The ROIs were drawn within 2 mm of the muscle boundaries. The differing area sizes (A_i) of the individual ROIs [ROI_i with individual fat fractions (FF_i)] were taken into account using the formula FF_mean_over_ROIs = sum (A_i ^∗^ FF_i)/sum (A_i), where the sum is the summation over all ROIs.

### Statistics

Group comparison and analysis of interrater agreement were performed. For group comparison, analysis was performed using the Mann–Whitney *U* test. Correlations were assessed by non-parametric Spearman correlation tests. Intraclass correlation coefficients (ICC) were considered indicative of interrater reliability. All tests were performed using dedicated software (Statistics Package for Social Sciences (SPSS), v26, IBM, Armonk, NY, United States and Graph Pad Prism, v7, GraphPad Software, San Diego, CA, United States). A *p*-value < 0.05 was considered statistically significant. Statistical analysis of the Graphs depict mean ± standard error of the mean.

## Results

### Demographics

There were no significant differences between the two cohorts in terms of the demographic data evaluated [sex, age, weight, body mass index (BMI)]. For a detailed comparison, see Table 1.

**TABLE 1:** Clinical data.

	**ALS**	**Controls**	***p* value**
Sex (female:male)	6:8	6:7	
Age (years)	62.6 (3.5)	56.9 (2.9)	0.06 (n.s.)
Height (cm)	172 (3.9)	179 (2.5)	0.28 (n.s.)
Weight (kg)	71.3 (4.0)	75.3 (4.68)	0.59 (n.s.)
BMI (cm/kg^2^)	24.1 (1.4)	25.4 (2.6)	0.92 (n.s.)
Therapy (Riluzole)	14/14		
Disease duration (years)	0.5 (0.1)		

### Clinical Characteristics of ALS Patients

The onset of disease occurred in the upper limbs in 50% of patients, the lower limbs in 28.57%, and the bulbar region in 21.42%. On average, the disease duration at the time of the examination was 4.8 ± 1 months. Mean ALSFRS-R score was 27.1 ± 1.77.

### TSE and DTI MRI Scans

**FIGURE 1:**
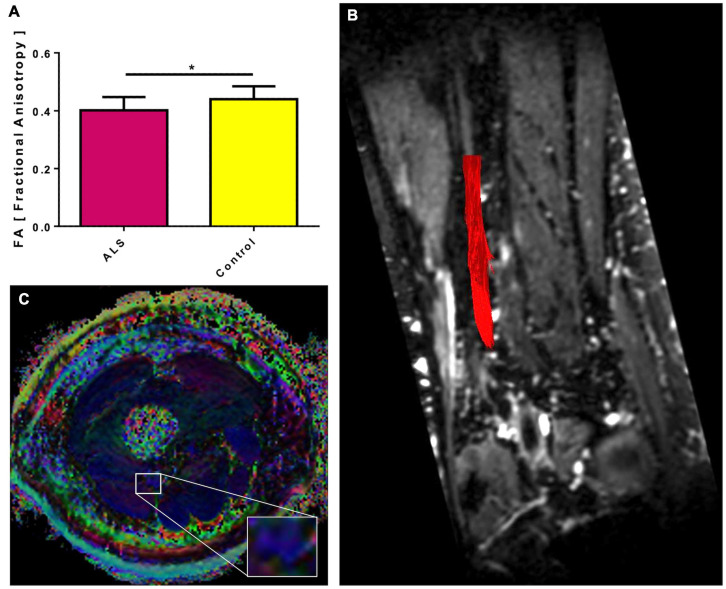
Fractional anisotropy (FA) of the sciatic nerve in ALS patients compared to controls. The sciatic nerves of ALS patients show a lower mean FA than healthy controls **(A)**. Significant differences are indicated by asterisks (*p < 0.05, **p < 0.01, ***p < 0.001). In **(B)**, for illustrative purposes, a deterministic fiber tracking of the sciatic nerve was projected in red color on a coronary reconstruction of the 3D T2 TSE sequence. In **(C)**, an exemplary color-coded FA map of an ALS patient is shown with the sciatic nerve magnified. The blue color of the nerve corresponds to a head-to-foot directional encoding.

The sciatic nerve was identified in all patients and controls on 3D and 2D T2 TSE MRI scans. DTI scans of the sciatic nerve in ALS patients showed significantly lower mean FA values than healthy controls. Mean values in ALS were 0.40 ± 0.012 (*p* = 0.025) compared to controls (0.44 ± 0.012) (Figure 1). Interrater reliability for DTI was excellent (ICC 0.873).

### PDFF Mapping

**FIGURE 2:**
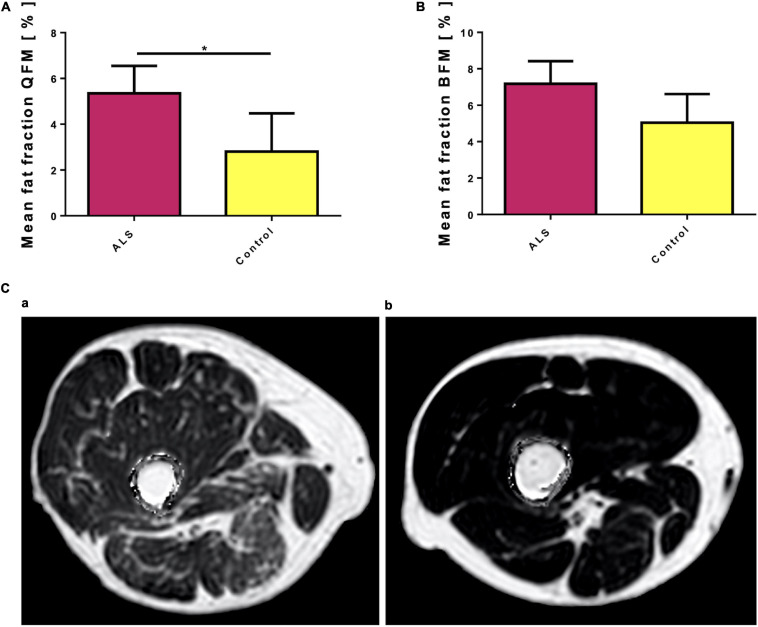
**(A)** Mean fat fraction of the quadriceps femoris muscle (QFM) in ALS patients compared to controls. The quadriceps femoris muscle of ALS patients shows a higher fat fraction compared to healthy controls. Significant differences are indicated by asterisks (*p < 0.05, **p < 0.01, ***p < 0.001). **(B)** Mean fat fraction of the biceps femoris muscle (BFM) in ALS patients compared to controls. The biceps femoris muscle of ALS patients shows a higher, but not significant higher fat fraction compared to healthy controls. **(C)** Two exemplary proton density fat fraction (PDFF) maps: Even in this rather severely affected patient (a), visually the increased intramuscular fat fraction of the investigated muscles (QFM and BFM) is hardly recognizable compared to a healthy control (b). However, quantitative evaluation of the Multi-echo Dixon showed increased intramuscular fat fractions for both muscle groups, although the difference was only significant for QFM. Note, that artifacts surrounding the thigh have been removed by software post-processing.

The quadriceps femoris muscle of ALS patients showed significantly higher intramuscular fat fractions than in healthy controls (mean 5.35 ± 1.2% vs. 2.8 ± 1.67, *p* = 0.038, Figure 2A). Intramuscular fat fractions in the biceps femoris muscle of ALS patients were also higher than in healthy controls, but this difference was not statistically different (mean 7.18 ± 1.24 vs. 5.03 ± 1.58, *p* = 0.155, Figure 2B). ROI sizes were between 14 and 2,133 mm^2^. Even in rather severely affected patients, the increased intramuscular fat fraction of the examined muscles is often hardly recognizable visually compared to healthy controls (Figure 2C). Interrater reliability for PDFF mapping was excellent (QFM: ICC 0.983, BFM: ICC 0.984).

No significant correlations were observed between the FA values of the sciatic nerve and ALSFRS-R (*r* = 0.12 *p* = 0.20), the FA values of the sciatic nerve and CMAP amplitude of the tibial and peroneal nerves (*r* = 0.2 and 0.42, *p* = 0.493 and 0.228), the FA values of the sciatic nerve and mean fat fraction of the BFM (*r* = −0.495 *p* = 0.072). A significant correlation was found between the FA values and mean fat fraction of the QFM in the ALS group (*r* = −0.578 *p* = 0.03). In the whole study population a significant correlation between FA and QFM as well as BFM could be established (r: −0.533, *p* = 0.004, r: −0.468, *p* = 0.014). Further correlations were found between QFM and BFM fat fraction all, HC and ALS and the whole study group (r: 0.753, *p* = 0.003, r: 0.807, *p* < 0.001, r: 0.835, *p* < 0.001). For a detailed list of electrophysiological data, see Table 2. Correlations between demographic and clinical data and the obtained imaging parameters are provided in Table 3.

**TABLE 2:** Electrophysiological data.

**Patient**	**Tibial nerve**		**Peroneal nerve**		**Sural nerve**	
	**NCV (m/s)**	**CMAP (mV)**	**NCV (m/s)**	**CMAP (mV)**	**NCV (m/s)**	**SNAP (μV)**
1	40	11.3	52	2.8	52	10
2	0	0	n.A.	n.A.	46	3
3	48	16.5	46	10.4	40	20
4	37	2.5	n.A.	n.A.	37	3
5	0	0	43	3.1	59	10
6	45	22.7	48	23.7	51	12
7	43	10.1	n.A.	n.A.	60	16
8	29	0.3	37	2.3	57	2
9	59	5.3	n.A.	n.A.	n.A.	n.A.
10	43	6.5	61	0.6	54	9
11	38.3	0.31	43.9	2.1	42	2.74
12	58.7	0.9	0	0	39.7	0.9
13	44	8.2	35	6.5	n.A.	n.A.
14	44	2.1	0	0	0	0

**TABLE 3:** Correlations between the collected demographic and clinical data and the obtained imaging parameters.

	**FA**	**QFM**	**BFM**
Age HC	−0.115 (*p* = 0.707)	0.5220 (*p* = 0.067)	0.401 (*p* = 0.174)
Age patients	0.007 (*p* = 0.982)	−0.020 (*p* = 0.946)	0.055 (*p* = 0.852)
Age both	−0.269 (*p* = 0.174)	0.342 (*p* = 0.080)	0.278 (*p* = 0.160)
Height HC	−0.626 (*p* = 0.022)*	−0.094 (*p* = 0.761)	0.174 (*p* = 0.570)
Height patients (12/14)	0.028 (*p* = 0.931)	−0.413 (*p* = 0.183)	−0.147 (*p* = 0.649)
Height both	−0.134 (*p* = 0.524)	−0.376 (*p* = 0.064)	−0.076 (*p* = 0.718)
Weight HC	−0.635 (*p* = 0.020)*	0.215 (*p* = 0.481)	0.580 (*p* = 0.038)*
Weight patients (12/14)	−0.615 (*p* = 0.033)*	0.294 (*p* = 0.354)	0.559 (*p* = 0.059)
Weight both	−0.547 (*p* = 0.005)**	0.191 (*p* = 0.359)	0.531 (*p* = 0.006)**
BMI HC	−0.225 (*p* = 0.459)	0.187 (*p* = 0.541)	0.434 (*p* = 0.138)
BMI patients (12/14)	−0.643 (*p* = 0.024)*	0.587 (*p* = 0.045)*	0.678 (*p* = 0.015)*
BMI both	−0.445 (*p* = 0.026)*	0.325 (*p* = 0.113)	0.555 (*p* = 0.004)**
NCV tibial nerve	0.265 (*p* = 0.361)	−0.743 (*p* = 0.002)**	−0.606 (*p* = 0.022)*
CMAP tibial nerve	0.200 (*p* = 0.493)	−0.275 (*p* = 0.341)	−0.231 (*p* = 0.427)
NCV peroneal nerve (10/14)	−0.091 (*p* = 0.802)	0.559 (*p* = 0.093)	0.454 (*p* = 0.089)
CMAP peroneal nerve (10/14)	0.419 (*p* = 0.228)	−0.219 (*p* = 0.544)	−0.103 (*p* = 0.776)
NCV sural nerve (12/14)	−0.448 (*p* = 0.145)	0.056 (*p* = 0.863)	0.364 (*p* = 0.245)
SNAP sural nerve (12/14)	0.126 (*p* = 0.696)	0.119 (*p* = 0.712)	0.270 (*p* = 0.396)
Disease duration	0.002 (*p* > 0.999)	0.134 (*p* = 0.649)	0.165 (*p* = 0.573)

Significant differences are in bold and indicated by asterisks (*p < 0.05, **p < 0.01, ***p < 0.001).

## Discussion

Our exploratory study evaluated the utility of a previously established MRI protocol for monitoring structural nerve damage in the sciatic nerve and intramuscular fat fraction of mid-thigh muscles. Our main findings are that ALS patients’ sciatic nerves demonstrate significantly lower FA values than healthy controls, and the thigh muscles in ALS patients accumulate more fat than thigh muscles from healthy controls. Although DTI derived FA in rodent (Takagi et al., 2009; Lehmann et al., 2010; Morisaki et al., 2011) and human peripheral nerves is considered a valid measure for axonal integrity and can be used to monitor axonal loss and regeneration as well as for demyelinating conditions (Sheikh, 2010; Kakuda et al., 2011; Mathys et al., 2013; Jende et al., 2020, 2021), data about FA values in peripheral nerves of ALS patients are sparse. In line with our findings, Simon and colleagues reported 8–10% lower FA values in the tibial and peroneal nerves in ALS patients than controls. We focused our study on a more proximal nerve segment based on previous experience (Lichtenstein et al., 2017). The sciatic nerve’s lower FA values indicate axon loss in our ALS patients cohort, thereby clearly distinguish them from healthy subjects. This finding is remarkable since the clinical data and the ALSFRS-R scores indicate that most patients were primarily affected in their upper extremities and did not show significant lower limb weakness. Our study confirms previous electrophysiologic studies that determined latencies of M- and H-responses in the lower extremity and found (indirect) evidence for proximal sciatic nerve damage (Koutlidis et al., 1984).

Compared to our previous findings, the decrease in FA values in ALS was much less pronounced compared to CIDP. This finding can be explained by the fact that most sciatic nerve fibers are sensory (Schmalbruch, 1986). It is tempting to speculate that changes in the FA values in ALS might be even bigger in motor nerves (i.e., the femoral nerve). Further studies are warranted to pursue this issue.

Furthermore, we provide additional data about the utility of PDFF mapping to quantify fatty infiltration in thigh muscles in ALS. In contrast to the visual impression, which was often rather subtle, the PDFF mapping showed higher muscle fatty infiltration in the ALS patients. Thereby, fatty infiltration was much more obvious in the quadriceps femoris muscle, but not in the biceps femoris muscle. Remarkably, there was a significant correlation between the FA values and the QFM fat fractions. Similar results were recently reported by Klickovic and colleagues. Our absolute values were almost twofold higher than those reported by a recent study that quantified fat accumulation in motor neuron disease, including ALS (Klickovic et al., 2019). These differences can be explained by different acquisition parameters, inter-MRI-vendor variability of PDFF mapping, and a difference in disease severity between the two examined cohorts. This issue emphasizes the importance of generating normal values and warrants caution regarding their interpretation when different acquisition soft- and hardware are used.

In this study, further correlations of minor relevance between the clinical or electrophysiological parameters and the measured image parameters or within the image parameters were also found. For example, significant correlations were found in parts between the intramuscular fat fractions of the examined muscles and the weight/BMI of the participants or in between the muscle groups.

This study has several limitations. First, we only included a small number of patients. Second, our data lacks follow-up examinations. Due to the pseudonymization process, a true blinding to groups (healthy vs. patients) was not feasible; however, readers were blinded to demographics as well as to detailed clinical history. Furthermore, we did not do systematic follow-up examinations. The assessment of multiple nerves, as mentioned above, especially motor nerves (e.g., femoral nerve) could provide even more specific results. The healthy controls tended to be of slightly younger age compared to the patients without revealing significant relevance; however, this needs to be considered as some studies showed that FA of the sciatic nerve is negatively associated with age in both healthy controls and patients with neuropathies (Kronlage et al., 2018; Jende et al., 2021). Of note, there was no significant correlation between age and FA values in our study cohort. Last, the use of further quantitative methods (e.g., T2 mapping) was omitted in favor of a time-optimized examination protocol.

In summary, we here present a multiparametric MRI protocol that allows non-invasive quantification of proximal structural nerve damage and muscle changes in ALS. We suggest that MR imaging of lower proximal limbs could be a valuable tool for quantifying the subclinical burden of axonal loss and neurogenic muscle changes in ALS. More extensive studies are justified to confirm its utility to serve as biomarkers in therapeutic trials.

## Data Availability Statement

The datasets presented in this article are not readily available due to data privacy reasons. Requests to access the datasets should be directed to TL, thorsten.lichtenstein@uk-koeln.de.

## Ethics Statement

The studies involving human participants were reviewed and approved by the Ethics Commission of the Faculty of Medicine of Cologne University. The patients/participants provided their written informed consent to participate in this study.

## Author Contributions

TL: study concept, conducting the study, analysis of data, data interpretation, and drafting the manuscript. AS: study concept, conducting the study, analysis of data, drafting the manuscript for content, review, and editing. KW: study concept, technical assistance, review, and editing. NG: analysis of data, review, and editing. DM, GF, and HL: study concept and drafting the manuscript for content. MS: data interpretation, drafting the manuscript for content, review, and editing. TH: study design and concept and drafting the manuscript. All authors contributed to the article and approved the submitted version.

## Conflict of Interest

NG is an editorial board member of European Radiology and a speaker at the bureau of Philips Healthcare; received research support from Philips Healthcare; and is a consultant for Bristol-Myers Squibb. GF serves as an editorial board member of Cortex, Neurological Research and Practice, NeuroImage: Clinical, Zeitschrift für Neuropsychologie, and DGNeurologie; receives royalties from the publication of the books Funktionelle MRT in Psychiatrie und Neurologie, Neurologische Differentialdiagnose, and SOP Neurologie; receives royalties from the publication of the neuropsychological tests KAS and Köpps; and received honoraria for speaking engagements from Bayer, Desitin, DGN, Ergo DKV, Forum für medizinische Fortbildung FomF GmbH, GSK, Medica Academy Messe Düsseldorf, Medicbrain Healthcare, Novartis, Pfizer, and Sportärztebund NRW. DM is a speaker at the bureau of Philips Healthcare. HL received honoraria for speaking and advisory board engagements or academic research support by Akcea, Alnylam, Biogen, Celgene, CSL Behring, Grifols, Gruenenthal, LFB Pharma, Takeda, and UCB. MS received personal fees from Bayer and Biogen. KW was employed by company Philips Healthcare. The remaining authors declare that the research was conducted in the absence of any commercial or financial relationships that could be construed as a potential conflict of interest.

## Publisher’s Note

All claims expressed in this article are solely those of the authors and do not necessarily represent those of their affiliated organizations, or those of the publisher, the editors and the reviewers. Any product that may be evaluated in this article, or claim that may be made by its manufacturer, is not guaranteed or endorsed by the publisher.
